# Supplementary material for: Patterns of Intron Gain and Loss in Fungi
Source: PLoS Biol. 2004 Nov 30;2(12):e422. doi: 10.1371/journal.pbio.0020422 (PMC532390; doi:10.1371/journal.pbio.0020422)
Supplement: Table S1 — Also available at http://genes.mit.edu/NielsenEtAl/. (4.3 MB ZIP). [file pbio.0020422.st001.zip › NielsenEtAl/html/1232.html]

AN3305.1.NCU01107.1.MG07443.1.FG07570.1


```
 CLUSTAL W (1.82) Multiple Sequence Alignments - Introns Inserted


Sequence 1: NCU01107.1	333 aa
Sequence 2: FG07570.1	334 aa
Sequence 3: AN3305.1	331 aa
Sequence 4: MG07443.1	320 aa
Alignment Length: 341 aa
Number Identitical Residues: 91 aa
Alignment Score (without introns) 5911


MG07443.1 	MSSLIPGSNTFNPD-KDIPDLSGK0HYIVTGGSAGIG~FGIAAHLLQHKADNVTILSNKE
NCU01107.1	MQSILQKATGIGPKPVQPTDLNGR~VAVVTGGAFGIG~FEVSRALANAGCR-VIMVNRKE
FG07570.1 	MQSILQKATGIGPQVIKPENLQGR~VAIVTGGALGIG~YEVSRALAHAGCK-VIMVNRKE
AN3305.1  	MQSVLQPI--IGPK-KTAEDLSGR~VAIITGGAFGIG2YEISRTFVQNGAR-VIMVNRKE
          	*.*::     :.*.     :*.*:   ::***: *** : ::  : :  .  * ::..**

MG07443.1 	EHAQSALEELKEYG-DASKAHWVKCDFEDLKFTEKVANELARSQDKLDG0LVLNAGLGVG
NCU01107.1	EQGDDAKATITAET-PGAVVEWRECDMGNLSQVRSVFSELREQLDRLDF~LVLSAGINTN
FG07570.1 	EQGTSAIEAVKNEK-HDADIDWKECDMGNLSQIREVFGGFRESLDRLDF~LVLSAGINTN
AN3305.1  	DQGQEAIDKIKEEAGSDAKIEWVPCDMGNLAQIREVASRFVEKEERLDL0LILSAGINVH
          	::. .*   :.   .  :  .*  **: :*   ..* . : .. ::**  *:*.**:.. 

MG07443.1 	VYNETKDKIDSHFQVNILSQFLLLKKLLPRLQKT------PGARLVFESSDLHKAAD-GG
NCU01107.1	QYGLDADGIDRHFGVNYLGQYYVVNQLYPVLKKTSKLPNTPAPRVVFEASEMHRAAP-SA
FG07570.1 	QFGLDSDGIERHFGVNFLGHFYVCNQLWPLLRKTSKMENVSPPRVVFEASEMHRLAQLSN
AN3305.1  	QYGETHDKIERHFQVNWLGQFYLTNLLWPLLRKTAKLPDTPAPRVVFESSEQHRAAP-SG
          	 :.   * *: ** ** *.:: : : * * *:**:.  . . .*:***:*: *: *  . 

MG07443.1 	AQFKDEAEINN-DIGPTQLYNRTKLALILLARALQRRKEAGELGFQPGQSVFINAVHPGA
NCU01107.1	VHFASLDEINNPDLGPTELYGRTKLAMILFAKYGLAEKVIKKN----GDNIYAASVHPGA
FG07570.1 	VQFKTKEEINDPDLDPTARYNRTKLAMILFAKYGLAGKVIAEN----KDKIYALSVHPGA
AN3305.1  	VKFGSLEEINNPDLGPLERYGRTKLAIILGVKYGLLEKVIKPN----GDNIYALSVHPGA
          	.:*    ***:.*:.*   *.*****:** .:     *          :.::  :*****

MG07443.1 	~VATDQQLQAEEAY-GTLGVVGHAIVRPFLKDPVSQGCRPALFATTSKEIEEQEISGQYI
NCU01107.1	0VNTAMQQQWKDAYPGITGKLLTWATLAFGRD-VKQGSYSALWALTDPKIEEQNLNGWYF
FG07570.1 	0VNTAMQQQWKDAYPGLTGKLLSWTMQAIGRD-VEQGSYSALWALTSSRIEEENLNGYYF
AN3305.1  	0VNTAMQQQWKDAYPGLFGKLLTTVMLAAGRN-VEQGSYSALYAAISPEVEEKGWNGYYL
          	 * *  * * ::**.*  * :      .  :: *.**. .**:*  . .:**:  .* *:

MG07443.1 	VPDKKVTSPSSKAQDDELGERLWNLMEQMLKDRLG----------
NCU01107.1	SDPDQPGKETSQASDPVLGQALWDLSERIIKDKLGEDALVDWYSA
FG07570.1 	VDPDKEGEETSQASNPELASNLWDLSTSFIKEKLGEDALADWNGS
AN3305.1  	KDVGQPGKESSLASDSVLGAALWDLSHRMIRDILGNDAVADWASA
          	    :  . :* *.:  *.  **:*   :::: **..:  .  .:
```
